# Supplementary material for: Sexual dimorphism in melanocyte stem cell behavior reveals combinational therapeutic strategies for cutaneous repigmentation
Source: Nat Commun. 2024 Jan 27;15:796. doi: 10.1038/s41467-024-45034-3 (PMC10821900; doi:10.1038/s41467-024-45034-3)
Supplement: Supplementary file 3 — Description of Additional Supplementary Files [file 41467_2024_45034_MOESM3_ESM.pdf]

Description of Additional Supplementary Files

File Name: Supplementary Data 1
Description: Supplemental Dataset 1 includes DESeq2 outputs of UV vs non-UV sample comparison in males and females; the list of differentially expressed genes that are significantly upregulated in males only, females only, and significantly upregulated in both sexes.
