# Supplementary material for: Sexual dimorphism in melanocyte stem cell behavior reveals combinational therapeutic strategies for cutaneous repigmentation
Source: Nat Commun. 2024 Jan 27;15:796. doi: 10.1038/s41467-024-45034-3 (PMC10821900; doi:10.1038/s41467-024-45034-3)
Supplement: Supplementary file 5 — Reporting Summary [file 41467_2024_45034_MOESM5_ESM.pdf]

nature portfolio

Corresponding author(s): Andrew C White

Last updated by author(s): Dec 19, 2023

# Reporting Summary

Nature Portfolio wishes to improve the reproducibility of the work that we publish. This form provides structure for consistency and transparency in reporting. For further information on Nature Portfolio policies, see our Editorial Policies and the Editorial Policy Checklist.

## Statistics

For all statistical analyses, confirm that the following items are present in the figure legend, table legend, main text, or Methods section.

| n/a | Confirmed | |
|---|---|---|
| ☐ | ☒ | The exact sample size (*n*) for each experimental group/condition, given as a discrete number and unit of measurement |
| ☐ | ☒ | A statement on whether measurements were taken from distinct samples or whether the same sample was measured repeatedly |
| ☐ | ☒ | The statistical test(s) used AND whether they are one- or two-sided<br>*Only common tests should be described solely by name; describe more complex techniques in the Methods section.* |
| ☒ | ☐ | A description of all covariates tested |
| ☒ | ☐ | A description of any assumptions or corrections, such as tests of normality and adjustment for multiple comparisons |
| ☒ | ☐ | A full description of the statistical parameters including central tendency (e.g. means) or other basic estimates (e.g. regression coefficient) AND variation (e.g. standard deviation) or associated estimates of uncertainty (e.g. confidence intervals) |
| ☐ | ☒ | For null hypothesis testing, the test statistic (e.g. *F*, *t*, *r*) with confidence intervals, effect sizes, degrees of freedom and *P* value noted<br>*Give P values as exact values whenever suitable.* |
| ☒ | ☐ | For Bayesian analysis, information on the choice of priors and Markov chain Monte Carlo settings |
| ☒ | ☐ | For hierarchical and complex designs, identification of the appropriate level for tests and full reporting of outcomes |
| ☐ | ☒ | Estimates of effect sizes (e.g. Cohen's *d*, Pearson's *r*), indicating how they were calculated |

*Our web collection on statistics for biologists contains articles on many of the points above.*

## Software and code

Policy information about availability of computer code

| | |
|---|---|
| Data collection | Melanocyte migration and Immunofluorescent images were collected using Leica LAS X V3.7.5. Live multiphoton image were collected using MATLAB SCANIMAGE. Flow cytometry data were collected using BD FACSDiva. |
| Data analysis | Immunofluorescent image analysis was perfomed using ImageJ V2.9.0, Imaris 9.0. Melanocyte quantification analysis was performed using code uploaded to figshare: https://figshare.com/projects/UVB-induced_melanocyte_stem_cell_activation/152487. Flow analysis were performed using FlowJo V10.8. BulkRNAseq were performed using Rstudio V2022.12.0+353. DEseq2 1.24.0. Single cell RNA seq analysis were performed using Cellranger and Seurat 4.3.0. |

For manuscripts utilizing custom algorithms or software that are central to the research but not yet described in published literature, software must be made available to editors and reviewers. We strongly encourage code deposition in a community repository (e.g. GitHub). See the Nature Portfolio guidelines for submitting code & software for further information.

## Data

Policy information about availability of data

All manuscripts must include a data availability statement. This statement should provide the following information, where applicable:

- Accession codes, unique identifiers, or web links for publicly available datasets
- A description of any restrictions on data availability
- For clinical datasets or third party data, please ensure that the statement adheres to our policy

The raw and processed bulk RNA sequencing data generated in this study have been deposited in the GEO database under accession code GSE247532 (https://www.ncbi.nlm.nih.gov/geo/query/acc.cgi?acc=GSE247532). The raw and processed scRNA seqeuncing data generated in this study have been deposited in the GEO database under accession code GSE247694 (https://www.ncbi.nlm.nih.gov/geo/query/acc.cgi?acc=GSE247694). The processed data, code for RNA sequencing analysis and MATLAB code for melanocyte migration analysis are available in figshare "UVB-induced melanocyte stem cell activation" (https://figshare.com/projects/UVB-induced_melanocyte_stem_cell_activation/152487). For bulk RNA seq, GRCm39 mouse reference genome was used. For scRNA seq, mm10 mouse reference genome were used.

## Research involving human participants, their data, or biological material

Policy information about studies with human participants or human data. See also policy information about sex, gender (identity/presentation), and sexual orientation and race, ethnicity and racism.

| | |
|---|---|
| Reporting on sex and gender | N/A |
| Reporting on race, ethnicity, or other socially relevant groupings | N/A |
| Population characteristics | N/A |
| Recruitment | N/A |
| Ethics oversight | N/A |

Note that full information on the approval of the study protocol must also be provided in the manuscript.

# Field-specific reporting

Please select the one below that is the best fit for your research. If you are not sure, read the appropriate sections before making your selection.

☒ Life sciences ☐ Behavioural & social sciences ☐ Ecological, evolutionary & environmental sciences

For a reference copy of the document with all sections, see nature.com/documents/nr-reporting-summary-flat.pdf

# Life sciences study design

All studies must disclose on these points even when the disclosure is negative.

| | |
|---|---|
| Sample size | Experiments were designed to have sufficient sample sizes to obtain reliable results. At least 4 animals were used in each group of comparison in most experiments with detected differences greater than 80% power and type I error rate of 5% using G*power V3.1.9.6. For experiments used with 3 animals, for example Fig5H, power analysis were used to confirm the power is above 99%. For experiments with mild differences (detected differences less than 80% power), for example Fig5I, 13 pairs of animals were used to achieve reliable results. Statistical analysis were performed using Prism 10. |
| Data exclusions | Animals in which dorsal skin went into anagen or showed skin burning due to UVB were excluded. |
| Replication | All experiments were replicated at least 3 times. Without specification, all individual data point in the manuscript indicate one biological replicate. Not all replication were successful due to the skin burning or anagen initiation issue described earlier. |
| Randomization | When using animals of the same genotype, animals for control and experiment groups were randomized. |
| Blinding | Analysis was double blinded. During data collection and analysis, only mouse ID were shown on raw data files. The genotype or ctrl/experiment group assignment behind each mouse ID were recorded in an independent document. Only melanocyte migration quantification between male and female were not blinded since it's obvious to distinguish male and female animals during mouse collections. |

# Reporting for specific materials, systems and methods

We require information from authors about some types of materials, experimental systems and methods used in many studies. Here, indicate whether each material, system or method listed is relevant to your study. If you are not sure if a list item applies to your research, read the appropriate section before selecting a response.

## Materials & experimental systems

| n/a | Involved in the study |
|---|---|
| ☐ | ☒ Antibodies |
| ☒ | ☐ Eukaryotic cell lines |
| ☒ | ☐ Palaeontology and archaeology |
| ☐ | ☒ Animals and other organisms |
| ☒ | ☐ Clinical data |
| ☒ | ☐ Dual use research of concern |
| ☒ | ☐ Plants |

## Methods

| n/a | Involved in the study |
|---|---|
| ☒ | ☐ ChIP-seq |
| ☐ | ☒ Flow cytometry |
| ☒ | ☐ MRI-based neuroimaging |

# Antibodies

Antibodies used

Flow cytometry antibodies:
CD45 (clone 30-F11; BD Biosciences Cat# 564279), CD11b (clone M1/70; Thermo Fisher Scientific Cat# 45-0112-80), CD115 (clone AFS98; Thermo Fisher Scientific Cat# 12-1152-81), Ly6C (clone HK1.4; Thermo Fisher Scientific Cat# 47-5932-80), Ly6G (clone 1A8, BD Biosciences Cat# 565369), F4/80 (clone BM8, Thermo Fisher Scientific Cat# 17-4801-80), Live/Dead Aqua or Violet fixable stains (Life: L34964, NC0180395).
IF staining antibodies:
F4/80 1:600 (Biolegend, Cat#123101); Ly6G 1:600 (Biolegend, Cat#127601); CD3 1:800 (Biolegend, Cat#100201); Cox-2 1:600 (Cayman, Cat#160106); Ptges2 1:200 (Abclonal, Cat#A7137); Ptges3 1:200 (Abclonal, Cat#A5194); Ptger2 1:500 (Abcam, Cat#ab167171); Cd49f 1:100 (BD Biosciences, Cat#555734); Dct 1:600 (Abcam, Cat#ab221144), Alex Fluor secondary antibodies (Abcam, Cat#ab150072, ab150149, ab150152, Fisher, Cat#A21207)

Validation

All the antibodies used in the study were bought from commercial vendors and were validated by the manufacturers:
CD45 BD:564279:Lagasse E, Connors H, Al-Dhalimy M, et al. Purified hematopoietic stem cells can differentiate into hepatocytes in vivo. Nat Med. 2000; 6(11):1212-1213.
CD11b Thermo:45-0112-80: Sasha Gupta et al. CAR-T Cell-Mediated B-Cell Depletion in Central Nervous System Autoimmunity. PMID: 36657993
CD115 Thermo:12-1152-81: M Christofi et al. Low-dose 2-deoxy glucose stabilises tolerogenic dendritic cells and generates potent in vivo immunosuppressive effects. PMID: 33074350
Ly6C: Thermo 12-1152-81: Shuai Guo et al. Gene-dosage effect of Pfkfb3 on monocyte/macrophage biology in atherosclerosis. PMID: 35834356
Ly6G BD: 565369: Wang JX, Bair AM, King SL, et al. Ly6G ligation blocks recruitment of neutrophils via a beta2-integrin-dependent mechanism. Blood. 2012; 120(7):1489-1498. (Biology).
F4/80 Thermo: 17-4801-80: Choi H et al. Lactate oxidase/catalase-displaying nanoparticles efficiently consume lactate in the tumor microenvironment to effectively suppress tumor growth.
F4/80 Biolegend: 123101: Radtke AJ, et al. 2020. Proc Natl Acad Sci U S A. 117:33455-65.
Ly6G Biolegend: 127601: Kobayashi A, et al. 2021. Front Immunol. 12:650856
CD3: Biolegend: 100201: Radtke AJ, et al. 2020. Proc Natl Acad Sci U S A. 117:33455-65. (SB)
Cox2 Cayman:160106: Kelleher, A.M., Milano-Foster, J., Behura, S.K., et al. Uterine glands coordinate on-time embryo implantation and impact endometrial decidualization for pregnancy success. Nat. Commun. 9(1), 2435 (2018).
Ptges2 Abclonal:A7137: Western blot analysis of extracts of various cell lines, using PTGES2 Rabbit pAb (A7137) at 1:1000 dilution by Abclonal.
Ptges3 Abclonal: A5194: Western blot analysis of extracts of various cell lines, using P23/PTGES3 Rabbit mAb (A5194) at 1:1000 dilution by Abclonal
Ptger2 Abcam:ab167171: Jiang W et al. PGE2 activates EP4 in subchondral bone osteoclasts to regulate osteoarthritis. Bone Res 10:27 (2022). PMID:35260562
Cd49f BD:555734: Aumailley M, Timpl R, Sonnenberg A. Antibody to integrin alpha 6 subunit specifically inhibits cell-binding to laminin fragment 8. Exp Cell Res. 1990; 188(1):55-60. (Biology).
Dct Abcam:ab221144: Ren S et al. Conjugate of ibrutinib with a TLR7 agonist suppresses melanoma progression and enhances antitumor immunity. Int J Biol Sci 18:166-179

# Animals and other research organisms

Policy information about studies involving animals; ARRIVE guidelines recommended for reporting animal research, and Sex and Gender in Research

Laboratory animals

Male and female mice with C57Bl/6 background and NSG immune-deficient mice. All mice were used between 7-10 weeks of age.

Wild animals

No wild animals were used in this study.

Reporting on sex

Yes, this study is focused on sex differences. Therefore, both sex have been reported and compared in this study.

Field-collected samples

No field-collected samples were used in this study.

| | |
|---|---|
| Ethics oversight | All animal experiments have been approved by the IACUC at Cornell University and all animal experiments were performed in animal facility at Cornell Animal Research and Education center. |

Note that full information on the approval of the study protocol must also be provided in the manuscript.

## Plants

| | |
|---|---|
| Seed stocks | N/A |
| Novel plant genotypes | N/A |
| Authentication | N/A |

## Flow Cytometry

### Plots

Confirm that:

☒ The axis labels state the marker and fluorochrome used (e.g. CD4-FITC).

☒ The axis scales are clearly visible. Include numbers along axes only for bottom left plot of group (a 'group' is an analysis of identical markers).

☒ All plots are contour plots with outliers or pseudocolor plots.

☒ A numerical value for number of cells or percentage (with statistics) is provided.

### Methodology

| | |
|---|---|
| Sample preparation | Sample preparation follows the standard methods published previously. Basically, fresh collected dorsal skin were digested into single cell suspension. Single cell suspension were stained with antibody cocktails for 30mins at 4C. After one time washing, single cell suspension were fixed for flow analysis. |
| Instrument | BD Symphony A3 |
| Software | FlowJo V10.8 |
| Cell population abundance | 300000 cells were analyzed for each sample |
| Gating strategy | Single cells were gated based on FSC and SSC. The other channel gatings were tested by FMO. |

☒ Tick this box to confirm that a figure exemplifying the gating strategy is provided in the Supplementary Information.
